# SERS for Detection of Proteinuria: A Comparison of Gold, Silver, Al Tape, and Silicon Substrates for Identification of Elevated Protein Concentration in Urine

**DOI:** 10.3390/s23031605

**Published:** 2023-02-01

**Authors:** Sultan Aitekenov, Alisher Sultangaziyev, Aigerim Boranova, Aigerim Dyussupova, Aisha Ilyas, Abduzhappar Gaipov, Rostislav Bukasov

**Affiliations:** 1 Department of Chemistry, School of Sciences and Humanities (SSH) Nazarbayev University, Nur-Sultan 010000, Kazakhstan; 2 Department of Medicine, School of Medicine, Nazarbayev University, Nur-Sultan 010000, Kazakhstan

**Keywords:** SERS, Raman spectroscopy, gold nanoparticles, urinary proteins, silicon, AUC, chronic kidney disease CKD

## Abstract

Excessive protein excretion in human urine is an early and sensitive marker of diabetic nephropathy and primary and secondary renal disease. Kidney problems, particularly chronic kidney disease, remain among the few growing causes of mortality in the world. Therefore, it is important to develop an efficient, expressive, and low-cost method for protein determination. Surface enhanced Raman spectroscopy (SERS) methods are potential candidates to achieve these criteria. In this paper, a SERS method was developed to distinguish patients with proteinuria from the healthy group. Commercial gold nanoparticles (AuNPs) with diameters of 60 nm and 100 nm, and silver nanoparticles (AgNPs) with a diameter of 100 nm were tested on the surface of four different substrates including silver and gold films, silicon, and aluminum tape. SERS spectra were acquired from 111 unique human urine samples prepared and measured for each of the seven different nanoparticle plus substrate combinations. Data analysis by the PCA-LDA algorithm and the ROC curves gave results for the diagnostic figures of merits. The best sensitivity, specificity, accuracy, and AUC were 0.91, 0.84, 0.88, and 0.94 for the set with 100 nm Au NPs on the silver substrate, respectively. Among the three metal substrates, the substrate with AuNPs and Al tape performed slightly worse than the other three substrates, and 100 nm gold nanoparticles on average produced better results than 60 nm gold nanoparticles. The 60 nm diameter AuNPs and silicon, which is about one order of magnitude more cost-effective than AuNPs and gold film, showed a relative performance close to the performance of 60 nm AuNPs and Au film (average AUC 0.88 (Si) vs. 0.89 (Au)). This is likely the first reported application of unmodified silicon in SERS substrates applied for direct detection of proteins in any biofluid, particularly in urine. These results position silicon and AuNPs@Si in particular as a perspective SERS substrate for direct urine analysis, including clinical diagnostics of proteinuria.

## 1. Introduction

Surface-enhanced Raman spectroscopy (SERS) is one of the most versatile and sensitive techniques available to scientists [1,2,3]. Compared to other popular techniques, such as fluorescence spectroscopy, Raman spectroscopy has an advantages of label-free, non-bleaching, relatively narrow peaks, opening the potential for multiplexed detection, including direct multiplexed detection, and versatility for the ability of detecting analytes from simple organic molecules to bacterial cells [4]. Since its discovery 40 years ago, this area of research has experienced a number of breakthroughs, namely in understanding the principles behind the phenomena [1]. This in turn has helped to produce higher and more reproducible Raman signal enhancements, properties that are essential for real-life applications.

The theory behind surface-enhancement of Raman scattering is mainly based on two complementary mechanisms, electromagnetic enhancement and chemical enhancement, which in combination produce a more than 10^6^ increase in the Raman signal. Electromagnetic enhancement arises from the light-induced electric fields on the surface of nanostructures. These enhanced electric fields are generated when the incident light is in resonance with the oscillations of conduction electrons of the metal nanoparticle, which will make conduction electrons oscillate collectively [5]. Electromagnetic enhancement increases the Raman scattering by at least a factor of 10^4^ and as high as 10^10^ [6,7]. On the other hand, chemical enhancement arises from the localized electronic resonance of the adsorbate or charge transfer resonance from the surface to the metal nanoparticles and gives an enhancement on the order of 10^2^–10^4^ [8]. With such high signal enhancements, the applications of SERS in biosensing and especially in clinical diagnostics have seen a significant increase in number and complexity [9]. Moreover, SERS can be integrated with other methods. For example, Xiang et al. showed how it was possible to simultaneously prepare an electrochemical sensor that also exhibited efficient and reproducible surface-enhanced Raman scattering activities for the probe molecules [10].

One of the most used media for clinical analyses is human urine. Tests based on the use of urine can detect a wide range of health issues, from cardiovascular and kidney illnesses to several types of cancer [11,12,13]. Urine is a complex biofluid consisting of hundreds of different molecules, which can be used as biomarkers for many clinical conditions [14]. Among them, excessive protein excretion, particularly albumin (urinary protein) excretion of 30 to 300 mg a day, which is called microalbuminuria, is an early and sensitive marker of diabetic nephropathy and cardiovascular and renal diseases [15,16]. These conditions are directly caused by chronic kidney disease (CKD) which in total accounts for almost 4.6% of all mortality [17]. Thus, the use of highly sensitive techniques such as SERS is highly beneficial for the early detection of protein concentration-related diseases such as CKD [18].

In our previous study involving gold nanoparticles on a gold film, we were able to prove the concept of protein level detection in proteinuria patients. We were able to achieve a sensitivity, a specificity, an accuracy, and an AUC of 0.976, 0.667, 0.833, and 0.905 for the set with 60 nm Au NPs, and 0.794, 0.886, 0.846, and 0.899 for the set with 100 nm Au NPs, respectively. In the present study, we probed silver, aluminum tape, and silicon as the SERS substrate materials for proteinuria detection. Silver is one of the most widely used SERS substrates, with which the original SERS effect was discovered and explained. However, it was reported that gold and silver film substrates are susceptible to corrosion, biodegradation and contamination with sulfur-containing compounds and hydrocarbons [19,20,21,22]. Al foil is a promising non-conventional substrates for SERS, which is not only easily and readily available in most places of the world, but it may also have better resistance to contamination and non-specific protein binding in bio-sensing applications [23]. Al foil has been reported as an SERS and SEF substrate in a number of sensing and bio-sensing applications [24,25,26,27]. Silicon has a lower cost and higher stability/resistance to contamination/oxidation in comparison to silver and gold substrates, which are prone to contamination/non-specific binding from S-containing compounds, including proteins, especially due to Au-S and Ag-S dative bonding [23]. Therefore, silicon has attracted increasing attention as a substrate for surface-enhanced spectroscopy for sensing and bio-sensing applications [27,28]. Bare silicon is very seldom used as a substrate for surface-enhanced Raman spectroscopy (SERS) [28]. However, Kaminska et al. showed that Au NPs on a silicon substrate could be a reproducible, sensitive, and stable SERS substrate for the analysis of complex biofluids including urine [29,30].

There are at least several reports where silicon has been used for SERS in combination with noble metals (e.g., Au and Ag), which are attached to silicon as a result of elaborate and costly procedures [31,32,33,34]. For instance, Kartashova et al. used gold-assisted chemical etching of silicon wafers to produce silicon nanowires, the tops of which were additionally modified with gold nanoparticles for label-free detection of bilirubin in artificial urine samples [35]. Another example is when Kaminska et al. prepared an SERS substrate where the silicon wafer surface was chemically modified in a multi-step procedure with long-chain silanes containing a thiol group capable of binding to gold nanoparticles [29].

However, commercial silicon wafers, chemically unmodified with any SAM to bind to gold nanoparticles, or silicon wafers uncoated with vacuum or electrochemically deposited gold or silver film/nanostructures are very rarely, if at all, reported as substrates for bioanalytical SERS applications. To the best of our knowledge, hereafter, we report for the first time a SERS substrate made of unmodified noble metal nanoparticles (commercial AuNPs) on an unmodified silicon wafer in any SERS clinically related application. In particular, we use this substrate for the direct analysis of urine, targeting the clinical diagnosis of proteinuria. This type of substrate is not only relatively simple to prepare, but also cost- and time-efficient which enables a wider range of potential applications in bio-sensing and clinical diagnostics.

In the present paper, we also increased the number of patients enrolled in our study from 78 to 112, which should further improve reliability of the presented results. In terms of the data analysis, we performed a similar procedure to the one described in our previous study, using background subtraction and spectra normalization followed by PCA-LDA (principal component analysis-linear discriminant analysis) analysis followed building an ROC curve [17]. PCA-LDA helps us to quantize our large amount of spectral data into a set of variables that can be further used to explain our results in terms of figures of merit such as sensitivity, specificity, accuracy, and others. This method was also used by other groups, for example, to detect breast cancer and prostate cancer [36,37,38]. Overall, in this paper, we explore the potential for the application of silver film, aluminum foil, and silicon wafers as alternatives to gold film-coated substrates for proteinuria detection by SERS. 

## 2. Materials and Methods

### 2.1. Samples

We used 112 urine samples collected by a hospital from each individual within a 24 h period. Those samples included urine from control (healthy) subjects and urine samples from patients with elevated content of protein (proteinuria): 53 individuals had a protein concentration below 300 mg/L, 59 patients had a protein concentration above 300 mg/L. Written consent from all patients was received. The hospital measured the protein concentration by a standard routine urinary test as well as urine volume, and concentrations of some other urine components (urea, etc.). All samples were stored at −20 °C.

### 2.2. Chemicals and Equipment

We applied suspensions of 60 nm- and 100 nm-diameter gold nanoparticles in PBS, which were purchased from Sigma-Aldrich (USA). Gold film (50 nm thickness)-coated test slides (3 × 1 inches) were purchased from EMF Dynasil (USA). Raman spectra were obtained with the confocal Raman micro-spectrometer The Horiba LabRam Evolution and AFM maps were obtained with AFM AIST-NT.

### 2.3. SEM and TEM Characterisation 

SEM images were obtained with a scanning electron microscope Zeiss Crossbeam540 (Oberkochen, Germany). The SEM imaging parameters were accelerating voltage: 5.00 kV, high resolution mode, working distance: 2.7 mm, current I probe: 52 pA, and an ESB grid = 833 V.

TEM was performed with a transmission electron microscope JEOL JEM—1400 Plus (Tokyo, Japan). The TEM measurement parameters were accelerating voltage: 80 kV, magnification: 200 K, and exposure time: 5.00 s

### 2.4. Substrate Preparation

The same procedure was used for the preparation of all substrates. We applied 60 nm Au NPs, 100 nm Au NPs, 100 nm Ag NPs on gold, silicon, and silver slides and slides covered by aluminum tape. These composite nanoparticles@metal film substrates were prepared by roughly the same method, which was described elsewhere [24,39]. Initially, a 1 mL suspension of commercial gold nanoparticles of 60 or 100 nm diameter (OD = 1.0) were centrifuged for 5 min at 3500× *g* and 1500× *g*, respectively, and the supernatant was removed and displaced with the similar amount of ultra-pure water. This cycle was repeated 3 times. An amount of 15 µL (microliters) of solution of gold nanoparticles was drop-casted onto the gold-coated test slides at room temperature and left to dry. Urine samples were thawed after storage in the freezer. Then, 15 µL of the urine sample was drop-casted onto the obtained solid spots from nanoparticles. Each sample was prepared in triplicate to maximize the reproducibility of the measurements.

### 2.5. Spectra Acquisition

Raman spectra were measured at 785 nm excitation, a magnification of ×10, a grating of 600 gr/mm, and a neutral density filter of 100% for all substrates. In the case of gold, silver, and aluminum tape substrates, the range was 400 to 1800 cm^−1^ and the acquisition time was 16 s. We took systematic, ensemble SERS measurements and spectra from 3 different spots were taken. The first spot was taken near the middle of a droplet, and the second and the third spots were (0, 200) and (200, 0) away from the first in the (x, y) axis, respectively, measured in micrometers. For each spot 12 spectra were averaged; therefore, 36 spectra were averaged for each urine sample.

The above-mentioned parameters were slightly altered for silicon substrates: the range was 600–1800 cm^−1^, the acquisition time was 30 s. We took 6 individual measurements from one spot, there were three spots in total; therefore, overall, 18 spectra were averaged for each urine sample.

### 2.6. Spectra Processing and Data Analysis

All spectra processing and data analyses were performed in Python by using its common libraries, such as Numpy, Scikit, Pandas, Scipy, and Regex. To remove the fluorescent background, we processed acquired spectra with ALS (asymmetric least squares algorithm). In order to remove outliers we applied a median filter and finally we applied Savitsky–Golay smoothing algorithm, which preserves intensities but removes unwanted features [40].

The processed spectra were analyzed by PCA-LDA (principal component analysis-discriminant analysis) method. The ROC (receiver operating characteristic) curves were obtained to build a statistical model. Using this model, we calculated diagnostic sensitivity, specificity, and accuracy. These are parameters are commonly reported as figures of merit in biomedical analysis/detection research papers. Sensitivity indicates how well a test can identify true positives results, specificity measures how well a test can identify true negatives, and accuracy shows how close or far off the measured results are from their true value [41]. Those parameters are calculated by the following formulas:

$$Sensitivity=\frac{TP}{P},\;Specificity=\frac{TN}{N},\;Accuracy=\frac{TP+TN}{P+N}$$

where *TP*, *TN*, *P*, and *N* are true positive, true negative, all positive, and all negative, respectively.

## 3. Results and Discussions

**Figure 1:**
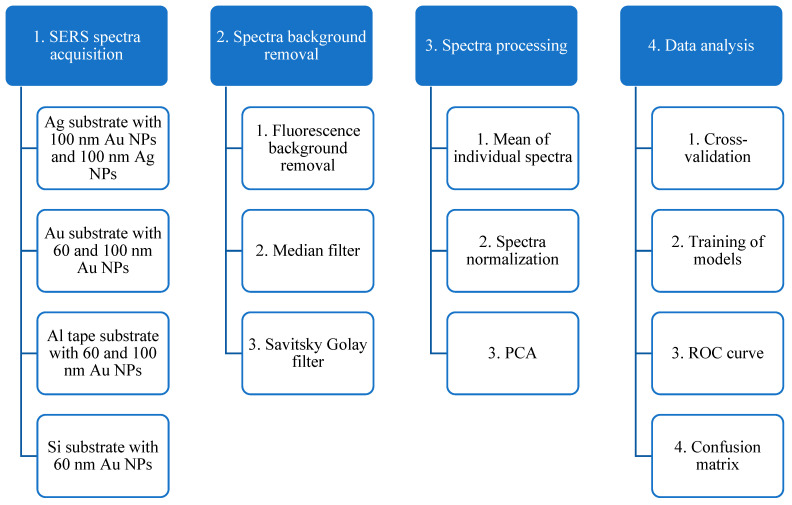
Employed analysis methods in the current work.

The goal of this work is to identify patients with high protein concentration and low protein group by taking their SERS spectra and performing further data analysis with the PCA-LDA algorithm. We measured the Raman spectra from various combinations of nanoparticles and substrates. All combinations and our methods for spectra processing and data analysis are presented in Figure 1. This work makes a significant advance over our previous work [17], both in experimental scope and data analysis. The objective of this study lies in a systematic assessment of diagnostic performance of various substrates, which is aimed to develop a method with better availability, good stability, and—even more importantly for their practical applications—significantly lower costs of the SERS substrates, while retaining relatively high standards of diagnostic accuracy.

**Figure 2:**
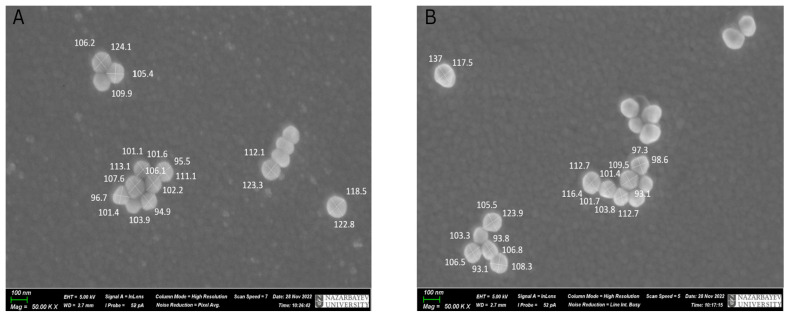
SEM pictures of 100 nm Ag NPs and 100 nm Au NPs on the silver substrates. (**A**) The SEM image of 100 nm Ag NPs with a magnification of 50,000. (**B**) The SEM image of 100 nm Au NPs with a magnification of 50,000.

Furthermore, the scanning electron microscopy (SEM) technique was used to analyze the size and distribution of the nanoparticles on the substrates. Figure 2A,B shows images of silver substrates with 100 nm Ag NPs and 100 nm Au NPs, obtained using an SEM microscope.

It was observed that nanoparticles have a relatively spherical shape and were distributed randomly on the substrate surface. In addition, urine samples were analyzed in terms of nanoparticle diameter by statistical analysis. The results showed that the mean and standard deviation were found to be 107 and 10 nm for 100 nm gold nanoparticles, and 107 and 7 nm for 100 nm silver nanoparticles. The mean and standard deviation were 72 and 10 nm for 60 nm gold nanoparticles. Single nanoparticles tended to agglomerate to form larger oligomers.

**Figure 3:**
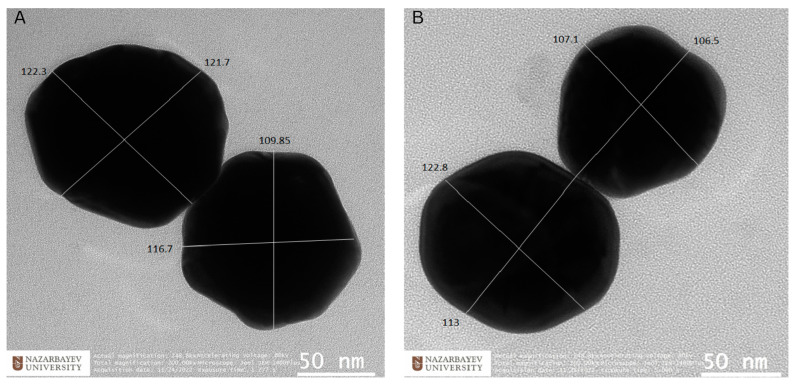
TEM images of 100 nm Au NPs and 100 nm Ag NPs. (**A**) The TEM image of 100 nm Au NPs with a magnification of 200,000. (**B**) The TEM image of 100 nm Ag NPs with a magnification of 200,000.

Figure 3A,B illustrates the pictures of 100 nm Ag NPs and 100 nm Au NPs, obtained using TEM imaging. Single nanoparticles tend to agglomerate to form larger oligomers. These aggregated clusters lead to enhanced SERS signals [42].

Additionally, the surface density was determined from SEM images. The surface density values were 3.53 ± 2.14 nanoparticles/µm^2^ for 60 nm Au NPs, 3.78 ± 2.07 nanoparticles/µm^2^ for 100 nm Au NPs, and 3.9 ± 3.81 nanoparticles/µm^2^ for 100 nm Ag NPs. It was proven that the area density and interparticle gap affect the SERS performance. A high surface density and a short distance between nanoparticles increase the number of active hot spots and improve the SERS enhancement factor [43]. For instance, SERS enhancement at the surface of the gold nanoparticle dimers on various substrates can be 1.5–2× (on gold, silver, and Al film) or even 10+× (on silicon) higher than SERS enhancement of single nanoparticles on the same substrate [25,26].

**Figure 4:**
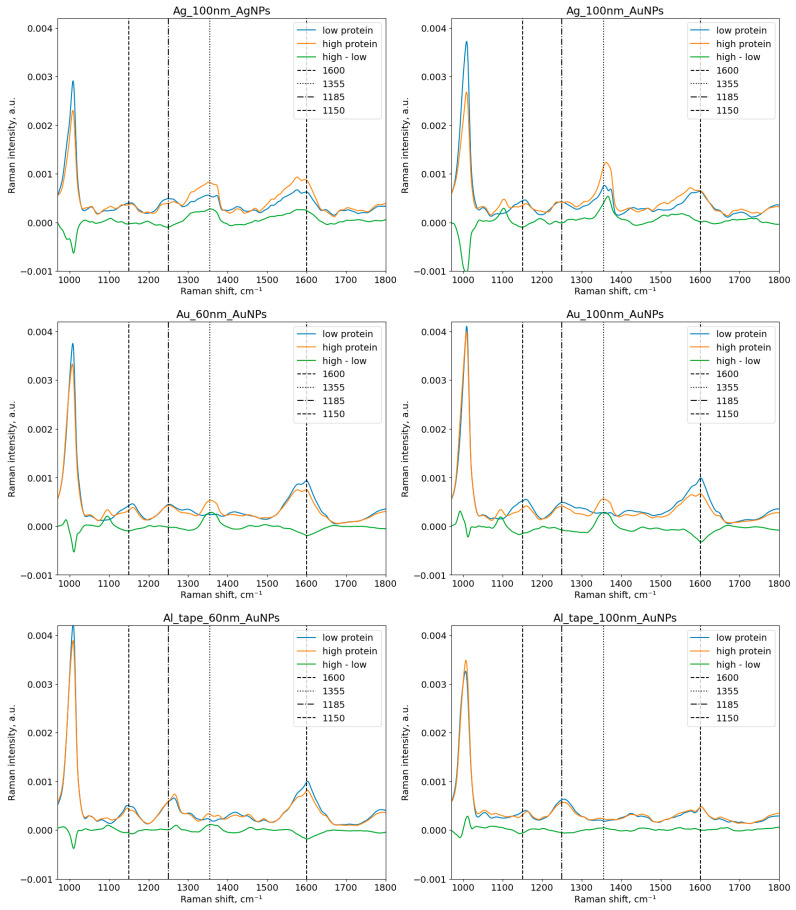
Average SERS spectra of urine samples with the low and the high protein groups with the spectrum of their difference for the metal substrates. The protein threshold is 300 mg/L. Au_100nm_AuNPs means 100 nm Au NPs on the gold substrate.

**Figure 5:**
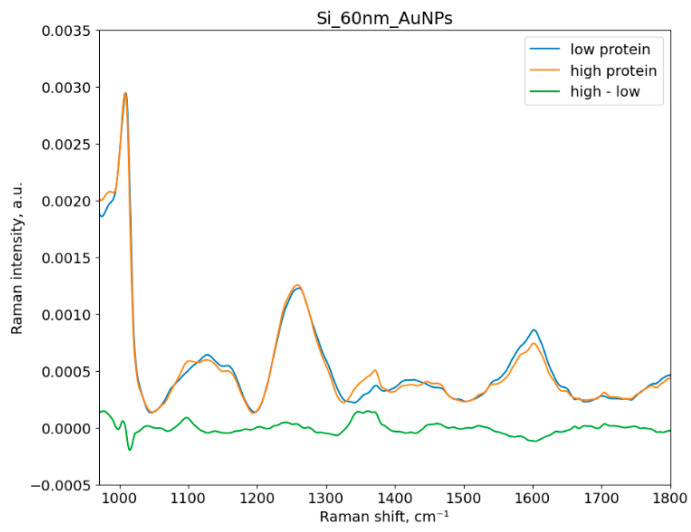
Average SERS spectra of urine samples with the low and the high protein groups with the spectrum of their difference for the silicon substrates. The protein threshold is 300 mg/L. Si_60nm_AuNPs means 60 nm Au NPs on the silicon substrate.

Figure 4 and Figure 5 show three averaged SERS spectra: one of urine samples with low protein concentration, another one of samples with high protein concentration, and the spectrum of their difference. The borderline between low/normal (for the control group) and elevated (for patients with proteinuria) protein concentration in urine is 300 mg/L of protein. The Raman shift range is 970–1800 cm^- 1^. Hereafter, we describe the band assignments in the obtained Raman spectra, which are based on the band assignments reported in the literature for SERS Raman spectra.

The peak at 1600–1700 cm^−1^ corresponds to H-O-H bending in water [44]. The peaks at 1400–1450 and 1050 cm^−1^ come from fatty acids [45]. The strongest peak in urine is 1005 cm^−1^ which corresponds to the N-C-N stretching in urea [46]. Certain peaks at 1359, 1554, and 1636 cm^−1^ can be attributed to specific amino acids [47]. For instance, the peaks at 1355, 1551, and 1610 cm^−1^ are attributed to tryptophan [48]. The peak related to albumin comes at 1450 cm^−1^ [49]. We have found that certain peaks can be associated with excess protein in human urine, such as the peaks at 1079, 1185, 1287, and 1383 cm^−1^ [50] and ~1150 and 1585 cm^−1^ [48,51]. Our experimental results are aligned with the mentioned papers. There are two strong peaks at ~1580–1600 and 1355 cm^−1^, and medium intensity peaks at 1185 and 1150 cm^−1^, as shown in Figure 4.

As mentioned in the Method Section, the discrimination between the low protein group and the high protein group was performed by application of PCA-LDA and ROC curves [52,53]. We set the protein concentration threshold to 300 mg/L. Historically, a protein excretion of more than 150 mg/day was regarded as abnormal [54]. However, the urine reagent strip devices that are commonly used in medical diagnostics have a higher threshold of about 300 mg/L [55], and we chose to pick the same threshold for SERS detection of protein urea as well. The relevant figures of merits are shown in Table 1 for the set with 100 nm Ag NPs on the silver substrate (Ag_100nm_AgNPs), which is one of the two best performing substrates.

**Figure 6:**
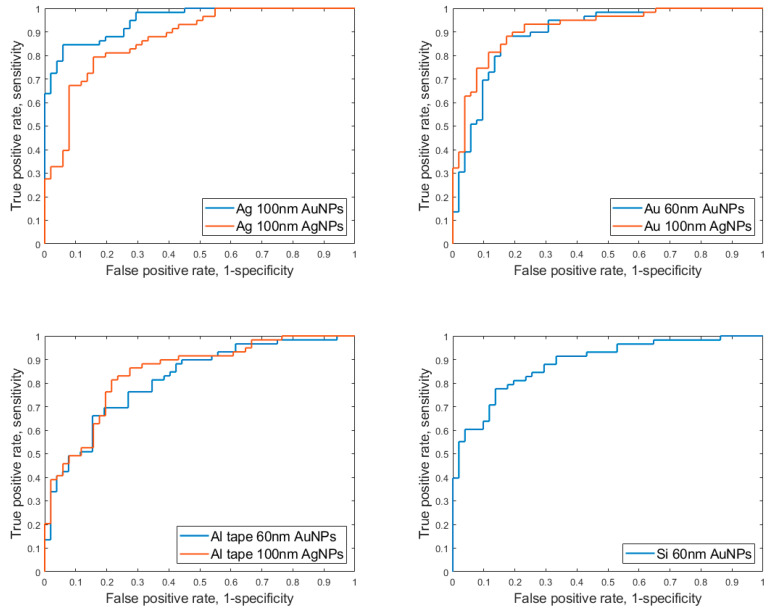
ROC curves for all experimental sets when PC components number is 25.

Similar tables on other experimental sets are given in the Supplementary Information. Table 2 shows the average of AUC values for each experimental set as a function of number of the PC components. The best parameters of sensitivity, specificity, accuracy, and AUC are given in Table 3. The parameters shown in Table 1 include sensitivity, specificity, accuracy, and confusion matrices and were calculated from respective ROC curves when the sum of sensitivity and specificity is maximized. A confusion matrix is a summary of prediction results on a classification problem that is divided into true positives (TP), true negatives (TN), false positives (FP), and false negatives (FN). Figure 6 demonstrates the ROC curves for all four substrates, which show relationships between sensitivity and specificity. The maximum sensitivity and specificity could be found from this curve, along with values for accuracy and values for a confusion matrix.

As seen from Table 1, figures of merits depend on the number of PC components. Particularly, AUC values greatly depend on multiple factors, and one of them is PC components. If we look solely at AUC vs. PC for an individual experimental set, AUC values are rising, but that results in overfitting data because AUC one-leave-out starts to decrease from certain PC values. The one-leave-out algorithm was used for avoiding the over-optimization of the model with excessive numbers of PC components. The average of two AUC values was used to construct Table 2. We believe that this average better represents performance of our experimental sets. Table 2 shows a clear outperformance of 100 nm NPs to 60 nm NPs in almost all instances by comparing either average or maximum values that are shown in the bottom rows.

If all experimental sets are compared by their maximum values of sum of AUC, we will have the following in descending order:

1. Silver substrate with 100 nm Au NPs (Ag_100nm_AuNPs);
2. Gold substrate with 100 nm Au NPs (Au_100nm_AuNPs);
3. Gold substrate with 60 nm Au NPs (Au_60nm_AuNPs);
4. Silicon substrate with 60 nm Au NPs (Si_60nm_AuNPs);
5. Silver substrate with 100 nm Ag NPs (Ag_100nm_AgNPs);
6. Aluminum tape with 100 nm Au NPs (Al_tape_100nm_AuNPs);
7. Aluminum tape with 60 nm Au NPs (Al_tape_60nm_AuNPs).

This order shows that 100 nm NPs perform better than 60 nm. This result was more or less expected. There are a number of works showing higher enhancement factor for nanoparticles with bigger size [17,34,35]. We would expect that increasing the gold nanoparticles particle size from 40 to 100 nm would produce a stronger LSPR for an excitation of 785 nm and produce larger SERS signals due to higher optical absorption and scattering of the substrates. Additionally, this order shows that if substrates with only 100 nm Au NPs are compared, the silver substrate is only slightly better than the gold substrate, and the gold substrate is better than the substrate with aluminum tape. The aluminum tape performs worse than the other substrates with both 60 and 100 nm Au NPs. This might be explained by relatively high diffuse reflectivity of Al tape surface, similar to a matte Al foil surface, which may contribute to a noisier background in the Raman spectra and therefore decrease the accuracy of data processing [56]. Additionally, as it was reported by Sergienko, an Al foil surface is expected to have a much higher surface roughness (25–30 nm vs. 3–4 nm) relative to the surface roughness of silicon, gold, and silver films used as SERS substrates in this study [26]. The variation in surface roughness is expected to have the same effect of less homogeneous SERS enhancement and higher variability in Raman spectra, decreasing overall accuracy. A possible way to improve the accuracy of a direct SERS assay of urine with an Al substrate, which we plan to attempt in the future, is to test substrates based on Al film or the glossy side of Al foil, which have significantly lower surface roughness and lower diffusive reflectivity. Comparable performance of the silicon substrate vs. gold substrate (similar average AUC of 0.79 and 0.80, respectively) is NOT surprising, since silicon has been reported as an efficiently good SERS active substrate in a number of works [57,58,59]. In fact, the lower non-specific binding of proteins to silicon is expected in comparison with non-specific binding to gold [23]. Therefore, protein molecules are less likely to diffuse away from the surface of AuNPs and particularly from the gaps between AuNPs—where most of the SERS enhancement is likely to occur—into the bare substrate, i.e., silicon. In other words, because of -S-Au dative bonding, proteins are more likely to stay on the more SERS enhancing surface of AuNPs when the substrate is silicon, if compared to the case when substrate is gold film. A similar affect may work for the AuNPs@silver film vs. the AuNPs@gold film, where the silver substrate may keep protein molecules better attached to nanogaps between AuNPs or just to the AuNPs surface, since the -S-Au bond is significantly stronger than the S-Ag bond [60]. Therefore, the relatively good performance of AuNPs@Ag film is also explainable.

Finally, we compared the performance of our method with the performance of other studies which have also analyzed or detect proteins or/and biomarkers in urine by Raman spectroscopy. We even looked for comparison at some other methods which have been used for detection of proteins/biomarkers in urine. The reported figures of merit of these studies used for comparison can be seen in Table 3. For example, a study by Zong et al. regarding the diagnosis of chronic kidney disease by SERS analysis of urine showed a slightly lower level of accuracy than the accuracy achieved in our research (82% vs. 86% average for 100 nm AuNPs on gold (0.847) and on silver (0.881) in the current paper) [50]. However, the accuracy calculated for our method of direct SERS of urine with AuNPs on silicon is about the same (82%) as accuracy reported by Zong et al. Moreover, our results were at least on par and frequently more accurate/better in comparison with results for the detection of proteinuria or CKD (or in one case a cancer marker) from other authors who employed various methods, reported by Rizk et al. [61], Zong et al. [50], Chock et al. [62], de Souza Vieira [63], Chen et al. [64], Chen et al. [65], and Satirapoj et al. [66]. For comparison, urinary dipstick tests, which are among the most common proteinuria diagnostic tests, demonstrate performance with a sensitivity of 73% and a specificity of 56% as reported by Nielsen et al. [67]. However, even our direct SERS analysis on 60 nm AuNPs@Si wafer achieves a sensitivity of 79% and a specificity of 84%. Additionally, the table suggests that the use of Raman spectroscopy and particularly SERS for urine analysis may have a comparable performance in detection across different diseases/conditions and thus there is a potential for simultaneous screening for different pathologies with comparable accuracy. Overall, we have obtained comparatively high AUC, accuracy, and other FOMs. Table 3 demonstrates the relative efficiency of this SERS method with gold nanoparticles on various substrates for the screening of proteinuria.

**Table 3:** Summary table of experimental papers on protein detection in human urine.

Paper	Method	Condition (Threshold)	Sensitivity	Specificity	Accuracy	AUC
**Current paper**	100 nm Au NPs@Au, SERS	Proteinuria 300 mg/L	0.932	0.750	0.847	0.897
	60 nm Au NPs@Au, SERS	Proteinuria 300 mg/L	0.915	0.769	0.847	0.893
	100 nm Au NPs@Ag, SERS	Proteinuria 300 mg/L	**0.914**	**0.843**	**0.881**	**0.941**
	100 nm Ag NPs@Ag, SERS	Proteinuria 300 mg/L	0.690	0.882	0.780	0.853
	100 nm Au NPs@Al_tape, SERS	Proteinuria 300 mg/L	0.814	0.784	0.800	0.844
	60 nm Au NPs@Al_tape, SERS	Proteinuria 300 mg/L	0.627	0.827	0.721	0.770
	60 nm Au NPs@Si, SERS	Proteinuria 300 mg/L	0.793	0.843	0.816	0.876
**Rizk et al.** [61]	SERS	Proteinuria	0.804	0.688	NA	0.82
**Zong et al.** [50]	SERS	Chronic kidney disease	0.78	0.86	0.818	0.886
**Chen et al.** [64]	Raman spectroscopy	Chronic renal failure	0.833	0.857	0.846	NA
**Nielsen et al.** [67]	Urinary dipstick test	Proteinuria	0.727	0.557	NA	0.762
**Chock et al.** [62]	Near infrared spectroscopy	Acute kidney injury	0.79	0.82	NA	NA
**De Souza Vieira et al.** [63]	Raman spectroscopy	Chronic kidney disease	NA	NA	0.79	NA
**Chen et al.** [65]	Liquid chromatography tandem mass spectrometry (LC–MS/MS)	Bladder cancer	0.711	0.75	NA	0.796
**Satirapoj et al.** [66]	ELISA immunoassay	Diabetic kidney disease	0.838	0.848	NA	0.84

Ideally, the maximum SERS enhancement factors are observed when the laser wavelength is shifted to the blue of the nanoparticle/nanostructure plasmon resonance and in the best case shifted by one-half of the Raman vibrational frequency [2,68].

Therefore, we have started working on a further improvement in direct (when no antibodies or aptamer are used to bind analyte) SERS application in protein analysis, which is the application of gold nanorods on gold or silicon as an SERS substrate. Since the localized surface plasmon resonance (LSPR) of gold nanorods (λ max is around 750 nm in suspension) is closer to the laser excitation wavelength of 785 nm, we expect higher enhancement with nanorods relative to SERS enhancement with gold nanoparticles, which have a λ max of around 570 nm in suspension for 100 nm diameter nanoparticles. Provided all other parameters of analysis are more or less equal, this higher enhancement may be translated to even more accurate results for clinical applications of direct SERS analysis.

## 4. Conclusions

The results show the potential of SERS spectroscopy in differentiating between patients with proteinuria and the healthy group for clinical diagnostics. The employed approach with three substrates and 60 nm and 100 nm NPs achieved good discrimination values as measured with AUC of the ROC curves. Data analysis with the PCA-LDA algorithm and the ROC curves gave results of diagnostic figures of merits. Using the threshold protein concentration of 300 mg/L, we obtained sensitivity, specificity, accuracy, and AUC values of 0.91, 0.84, 0.88, and 0.94, respectively, for the measurements on the 100 nm gold nanoparticles (AuNPs) on the silver substrate. Among three metal substrates, the substrate with aluminum tape performed the worst, while the performances of the gold and silver substrates were about the same. The silicon substrate performed nearly as well as noble metal (Au and Ag) substrates. The results obtained with 100 nm AuNPs appeared to be better when compared to the results obtained with 60 nm AuNPs on three substrates, i.e., gold, silver, and aluminum.

Overall, the 100 nm-diameter gold nanoparticles on silver film measured with 785 nm laser excitation appear as the optimal combination, providing the best balance between sensitivity and specificity in this SERS-based method of proteinuria diagnostics. The performance of the assay on silicon is also very promising and this substrate may provide an optimal combination of good performance with relatively low price, or the best “bang for buck” among the four SERS substrate materials tested for proteinuria detection. In fact, commercial gold nanoparticles or gold nanorods, which are likely to be tested in the future, set on unmodified silicon, may bring the cost of the substrate down by at least several times when compared to the cost of nanoparticles on gold film. The data in the Supplementary Materials show that for Au vs. Si substrates, the cost ratio of substrate materials, including the cost of 60 nm AuNPs, is 6.5. Therefore, the development of this kind of SERS substrate on silicon can make the practical clinical application of SERS for direct protein analysis far more likely.

## Figures and Tables

**Table 1:** Summary table for the set with 100 nm Ag NPs on the silver substrate.

PC Components	Total Variance Explained, %	Sensitivity	Specificity	Accuracy	AUC	AUC One-Leave-Out	AUC Sum	TP	TN	FP	FN	No
**5**	79.977	0.8136	0.6923	0.7568	0.7973	0.7448	1.5420	48	36	16	11	111
**7**	85.430	0.6102	0.9231	0.7568	0.8067	0.7422	1.5489	36	48	4	23	111
**9**	88.336	0.7797	0.8654	0.8198	0.8638	0.7937	1.6574	46	45	7	13	111
**11**	90.423	0.7627	0.8654	0.8108	0.8651	0.7839	1.6490	45	45	7	14	111
**13**	91.933	0.9322	0.7500	0.8468	0.8973	0.7940	1.6913	55	39	13	4	111
**15**	93.078	0.8136	0.8654	0.8378	0.8996	0.7859	1.6855	48	45	7	11	111
**17**	94.007	0.7627	0.9231	0.8378	0.8999	0.7686	1.6685	45	48	4	14	111
**19**	94.784	0.8475	0.9038	0.8739	0.9113	0.7647	1.6760	50	47	5	9	111
**21**	95.394	0.8475	0.8846	0.8649	0.9087	0.7500	1.6587	50	46	6	9	111
**23**	95.930	0.8305	0.9038	0.8649	0.9094	0.7317	1.6411	49	47	5	10	111
**25**	96.398	0.8814	0.8269	0.8559	0.9156	0.7076	1.6232	52	43	9	7	111

**Table 2:** Average of AUC values for each experimental set compared to PC components.

PC Components	Au_100nm_AuNPs	Au_60nm_AuNPs	Ag_100nm_AuNPs	Ag_100nm_AgNPs	Al_tape_100nm_AuNPs	Al_tape_60 nm_ AuNPs	Si_60nm_AuNPs
**5**	0.7710	0.7112	0.7649	0.6423	0.5150	0.6315	0.6202
**7**	0.7744	0.6952	0.7928	0.6859	0.5623	0.6506	0.6912
**9**	0.8287	0.7016	0.8612	0.7338	0.5924	0.7181	0.6805
**11**	0.8245	0.7236	0.8693	0.7375	0.5891	0.7091	0.7128
**13**	0.8457	0.7518	0.8643	0.7373	0.7004	0.7062	0.7818
**15**	0.8427	0.7516	0.8641	0.7273	0.6889	0.6957	0.7863
**17**	0.8343	0.7555	0.8687	0.7336	0.6982	0.6866	0.7836
**19**	0.8380	0.7686	0.8587	0.7703	0.7208	0.6734	0.7720
**21**	0.8294	0.7795	0.8746	0.7610	0.7164	0.6701	0.7912
**23**	0.8206	0.8049	0.8648	0.7606	0.7232	0.6729	0.7826
**25**	0.8116	0.7999	0.8580	0.7593	0.7295	0.6871	0.7796
max	**0.8457**	**0.8049**	**0.8746**	**0.7703**	**0.7295**	**0.7181**	**0.7912**
relative performance	**118**	**112**	**122**	**107**	**102**	**100**	**110**

Ag_100nm_AgNPs means 100 nm Ag NPs on the silver substrate.

## Supplementary Materials

The following supporting information can be downloaded at: https://www.mdpi.com/article/10.3390/s23031605/s1.

## Abbreviations

SERS	surface-enhanced Raman spectroscopy
PCA	principal component analysis
PC	principal component
LDA	linear discriminant analysis
AUC	area under the (ROC) curve
SEM	scanning electron microscope
TEM	transmission electron microscope
