# Supplementary material for: SERS for Detection of Proteinuria: A Comparison of Gold, Silver, Al Tape, and Silicon Substrates for Identification of Elevated Protein Concentration in Urine
Source: Sensors (Basel). 2023 Feb 1;23(3):1605. doi: 10.3390/s23031605 (PMC9921516; doi:10.3390/s23031605)
Supplement: Supplementary file 1 [file sensors-23-01605-s001.zip › sensors-2118621-supplementary.pdf]

Supplementary Information

SERS for Detection of Proteinuria: A Comparison of Gold, Silver, Al Tape, and Silicon Substrates for Identification of Elevated Protein Concentration in Urine

Sultan Aitekenov [1], Alisher Sultangaziyev [1], Aigerim Boranova [1], Aigerim Dyussupova [1], Aisha Ilyas [1], Abduzhappar Gaipov [2] and Rostislav Bukasov [1,*]

[1] Department of Chemistry, School of Sciences and Humanities (SSH) Nazarbayev University, Nur-Sultan 010000, Kazakhstan
2 Department of Medicine, School of Medicine, Nazarbayev University, Nur-Sultan 010000, Kazakhstan
* Correspondence: rostislav.bukasov@nu.edu.kz

SEM and TEM

Table S1. Calculation of surface density of nanoparticles using SEM images.

Nanoparticles	SEM image magnification	Number of nanoparticles, N	Area of surface, µm2	Surface density, N/µm2
60 nm AuNPs	x50000	19	3.32	5.72
	x50000	22	3.32	6.63
	x50000	23	3.32	6.93
	x50000	9	3.32	2.71
	x50000	13	3.32	3.92
	x50000	8	3.32	2.41
	x20000	35	20.02	1.75
	x20000	44	20.02	2.20
	x20000	39	20.02	1.95
	x20000	22	20.02	1.10
			**Mean**	**3.53**
			**Standard deviation**	**2.14**
100 nm AuNPs	x50000	19	3.32	5.72
	x50000	10	3.32	3.01
	x50000	16	3.32	4.82
	x50000	24	3.32	7.23
	x50000	12	3.32	3.61
	x50000	18	3.32	5.42
	x20000	43	20.02	2.15
	x20000	77	20.02	3.85
	x20000	18	20.02	0.90
	x20000	21	20.02	1.05
			**Mean**	**3.78**
			**Standard deviation**	**2.07**
	x50000	30	3.32	9.04
100 nm AgNPs	x50000	15	3.32	4.52
	x20000	27	20.02	1.35
	x20000	14	20.02	0.70
			**Mean**	**3.90**
			**Standard deviation**	**3.81**

Table S2. Surface density of nanoparticles.

Nanoparticles	Total number of nanoparticles, N	Surface density, N/µm2	Standard deviation
60 nm AuNPs	234	3.53	2.14
100 nm AuNPs	258	3.78	2.07
100 nm AgNPs	86	3.90	3.81

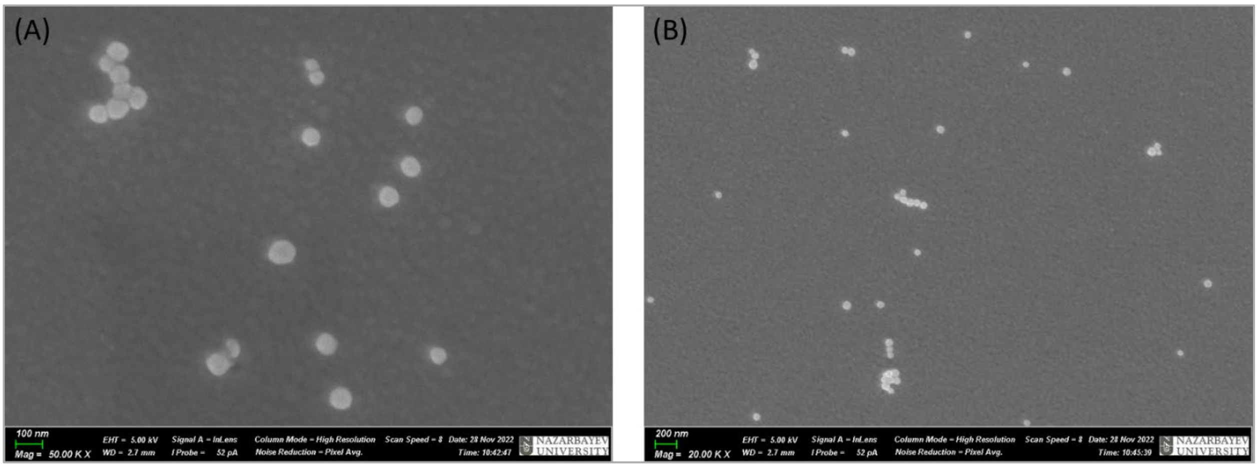

Figure S1. SEM pictures of 60 nm Au NPs on the silver substrates. (**A**) The SEM image of 60 nm Au NPs with magnification of 50,000. (**B**) The SEM image of 60 nm Au NPs with magnification of 20,000.

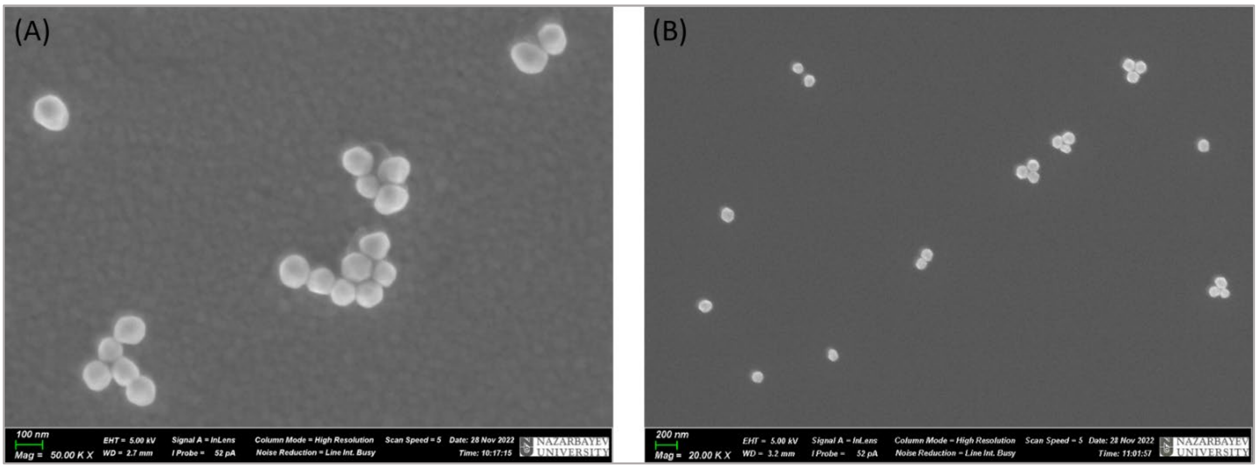

Figure S2. Representative SEM images of 100 nm Au NPs on the silver substrates. (**A**) The SEM image of 100 nm Au NPs with magnification of 50,000. (**B**) The SEM image of 100 nm Au NPs with magnification of 20,000.

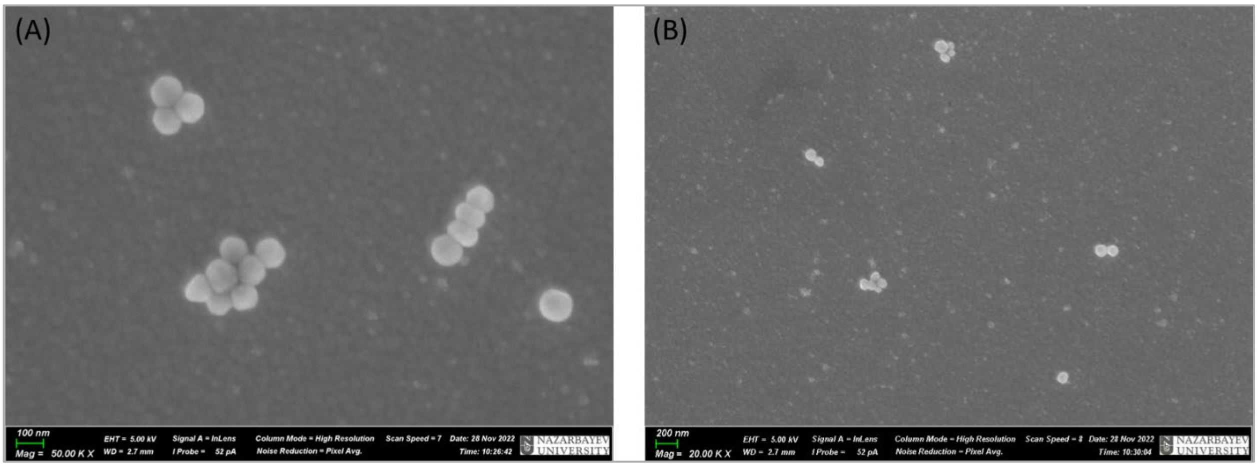

Figure S3. Representative SEM images of 100 nm Ag NPs on the silver substrates. (**A**) The SEM image of 100 nm Ag NPs with magnification of 50,000. (**B**) The SEM image of 100 nm Ag NPs with magnification of 20,000.

Table S3. Results for 100 nm AuNPs @ Au film .

PC component	Total variance explained,	Sens	Spec	Acc	AUC	AUC one-leave-out	AUC sum	TP	TN	FP	FN	No
5	80.0	0.81	0.69	0.76	0.80	0.74	0.771	48	36	16	11	111
7	85.4	0.61	0.92	0.76	0.81	0.74	0.774	36	48	4	23	111
9	88.3	0.78	0.87	0.82	0.86	0.79	0.829	46	45	7	13	111
11	90.4	0.76	0.87	0.81	0.87	0.78	0.824	45	45	7	14	111
13	91.9	0.93	0.75	0.85	0.90	0.79	0.846	55	39	13	4	111
15	93.1	0.81	0.87	0.84	0.90	0.79	0.843	48	45	7	11	111
17	94.0	0.76	0.92	0.84	0.90	0.77	0.834	45	48	4	14	111
19	94.8	0.85	0.90	0.87	0.91	0.76	0.838	50	47	5	9	111
21	95.4	0.85	0.88	0.86	0.91	0.75	0.829	50	46	6	9	111
23	95.9	0.83	0.90	0.86	0.91	0.73	0.821	49	47	5	10	111
25	96.4	0.88	0.83	0.86	0.92	0.71	0.812	52	43	9	7	111

Sens = Sensitivity, Spec = specificity, Acc = accuracy, AUC = area under the curve, TP = true positive, TN = true negative FP = false positive , FN = false negative, No = total number of unique patient samples, AUC sum is average of AUC and AUC one-leave-out.

Table S4. Results for 60 nm AuNPs @ Au film .

PC components	Total variance explained, %	Sensitivity	Specificity	Accuracy	AUC	AUC one-leave-out	AUC sum	TP	TN	FP	FN	No
5	76.3	0.61	0.85	0.72	0.75	0.67	0.71	36	44	8	23	111
7	83.7	0.61	0.88	0.74	0.75	0.65	0.70	36	46	6	23	111
9	86.9	0.63	0.85	0.73	0.76	0.64	0.70	37	44	8	22	111
11	89.1	0.66	0.85	0.75	0.78	0.66	0.72	39	44	8	20	111
13	90.7	0.73	0.83	0.77	0.82	0.69	0.75	43	43	9	16	111
15	92.1	0.66	0.90	0.77	0.83	0.68	0.75	39	47	5	20	111
17	93.3	0.78	0.79	0.78	0.84	0.67	0.76	46	41	11	13	111
19	94.2	0.85	0.79	0.82	0.85	0.68	0.77	50	41	11	9	111
21	95.0	0.86	0.81	0.84	0.87	0.69	0.78	51	42	10	8	111
23	95.6	0.92	0.77	0.85	0.89	0.72	0.80	54	40	12	5	111
25	96.1	0.88	0.83	0.86	0.90	0.70	0.80	52	43	9	7	111

Table S5. Results for 100 nm AgNPs @ Au film .

PC components	Total variance explained, %	Sensitivity	Specificity	Accuracy	AUC	AUC one-leave-out	AUC sum	TP	TN	FP	FN	No
5	85.8	0.66	0.84	0.74	0.79	0.74	0.76	38	43	8	20	109
7	90.0	0.91	0.67	0.80	0.82	0.77	0.79	53	34	17	5	109
9	92.3	0.79	0.86	0.83	0.89	0.83	0.86	46	44	7	12	109
11	93.7	0.81	0.86	0.83	0.91	0.83	0.87	47	44	7	11	109
13	94.7	0.86	0.82	0.84	0.91	0.82	0.86	50	42	9	8	109
15	95.5	0.86	0.86	0.86	0.91	0.81	0.86	50	44	7	8	109
17	96.1	0.84	0.90	0.87	0.93	0.81	0.87	49	46	5	9	109
19	96.5	0.84	0.86	0.85	0.92	0.79	0.86	49	44	7	9	109
21	97.0	0.91	0.84	0.88	0.94	0.81	0.87	53	43	8	5	109
23	97.3	0.88	0.90	0.89	0.95	0.78	0.86	51	46	5	7	109
25	97.6	0.84	0.94	0.89	0.95	0.77	0.86	49	48	3	9	109

AUC = area under the curve, TP = true positive, TN = true negative FP = false positive, FN = false negative, No = total number of unique patient samples , AUC sum is average of AUC and AUC one-leave-out.

Table S6. Results for 100 nm AgNPs @ Ag film .

PC components	Total variance explained, %	Sensitivity	Specificity	Accuracy	AUC	AUC one-leave-out	AUC sum	TP	TN	FP	FN	No
5	87.7	0.79	0.57	0.69	0.69	0.59	0.64	46	29	22	12	109
7	90.9	0.71	0.69	0.70	0.73	0.64	0.69	41	35	16	17	109
9	93.0	0.90	0.59	0.75	0.78	0.69	0.73	52	30	21	6	109
11	94.4	0.59	0.86	0.72	0.80	0.68	0.74	34	44	7	24	109
13	95.5	0.91	0.57	0.75	0.80	0.67	0.74	53	29	22	5	109
15	96.2	0.76	0.71	0.73	0.80	0.65	0.73	44	36	15	14	109
17	96.8	0.91	0.59	0.76	0.82	0.65	0.73	53	30	21	5	109
19	97.2	0.69	0.88	0.78	0.85	0.69	0.77	40	45	6	18	109
21	97.6	0.88	0.69	0.79	0.86	0.67	0.76	51	35	16	7	109
23	97.9	0.81	0.78	0.80	0.87	0.65	0.76	47	40	11	11	109
25	98.2	0.79	0.84	0.82	0.87	0.64	0.76	46	43	8	12	109

Table S7. Results for 100 nm AuNPs @ Al tape.

PC components	Total variance explained, %	Sensitivity	Specificity	Accuracy	AUC	AUC one-leave-out	AUC sum	TP	TN	FP	FN	No
5	76.4	0.44	0.76	0.59	0.60	0.43	0.51	26	39	12	33	110
7	83.0	0.63	0.69	0.65	0.63	0.49	0.56	37	35	16	22	110
9	86.7	0.76	0.59	0.68	0.67	0.52	0.59	45	30	21	14	110
11	89.1	0.66	0.67	0.66	0.68	0.50	0.59	39	34	17	20	110
13	90.8	0.69	0.75	0.72	0.77	0.63	0.70	41	38	13	18	110
15	92.1	0.69	0.75	0.72	0.77	0.61	0.69	41	38	13	18	110
17	93.2	0.58	0.88	0.72	0.79	0.60	0.70	34	45	6	25	110
19	94.1	0.64	0.88	0.75	0.82	0.62	0.72	38	45	6	21	110
21	94.8	0.68	0.86	0.76	0.83	0.60	0.72	40	44	7	19	110
23	95.3	0.80	0.76	0.78	0.83	0.61	0.72	47	39	12	12	110
25	95.9	0.81	0.78	0.80	0.84	0.61	0.73	48	40	11	11	110

AUC = area under the curve, TP = true positive, TN = true negative FP = false positive, FN = false negative, No = total number of unique patient samples, AUC sum is average of AUC and AUC one-leave-out.

Table S8. Results for 60 nm AuNPs @ Al tape.

PC components	Total variance explained, %	Sensitivity	Specificity	Accuracy	AUC	AUC one-leave-out	AUC sum	TP	TN	FP	FN	No
5	78.8	0.68	0.63	0.66	0.68	0.58	0.63	40	33	19	19	111
7	85.3	0.83	0.54	0.69	0.71	0.59	0.65	49	28	24	10	111
9	88.6	0.63	0.83	0.72	0.77	0.67	0.72	37	43	9	22	111
11	90.8	0.63	0.85	0.73	0.77	0.64	0.71	37	44	8	22	111
13	92.2	0.71	0.81	0.76	0.78	0.63	0.71	42	42	10	17	111
15	93.4	0.68	0.85	0.76	0.78	0.61	0.70	40	44	8	19	111
17	94.3	0.56	0.94	0.74	0.78	0.59	0.69	33	49	3	26	111
19	95.0	0.58	0.92	0.74	0.78	0.56	0.67	34	48	4	25	111
21	95.6	0.56	0.90	0.72	0.78	0.56	0.67	33	47	5	26	111
23	96.2	0.63	0.88	0.75	0.80	0.55	0.67	37	46	6	22	111
25	96.6	0.66	0.85	0.75	0.82	0.56	0.69	39	44	8	20	111

Table S9. Results for 60 nm AuNPs @ Silicon.

PC components	Total variance explained, %	Sensitivity	Specificity	Accuracy	AUC	AUC one-leave-out	AUC sum	TP	TN	FP	FN	No
5	86.1	0.41	0.86	0.62	0.67	0.57	0.620	24	44	7	34	109
7	91.0	0.74	0.69	0.72	0.73	0.65	0.691	43	35	16	15	109
9	93.4	0.53	0.86	0.69	0.74	0.62	0.681	31	44	7	27	109
11	94.6	0.81	0.69	0.75	0.78	0.65	0.713	47	35	16	11	109
13	95.4	0.83	0.71	0.77	0.84	0.73	0.782	48	36	15	10	109
15	96.0	0.72	0.84	0.78	0.85	0.72	0.786	42	43	8	16	109
17	96.5	0.74	0.82	0.78	0.85	0.72	0.784	43	42	9	15	109
19	96.9	0.72	0.86	0.79	0.86	0.69	0.772	42	44	7	16	109
21	97.2	0.79	0.84	0.82	0.88	0.71	0.791	46	43	8	12	109
23	97.5	0.79	0.84	0.82	0.88	0.69	0.783	46	43	8	12	109
25	97.8	0.78	0.86	0.82	0.89	0.67	0.780	45	44	7	13	109

AUC = area under the curve, TP = true positive, TN = true negative FP = false positive, FN = false negative, No = total number of unique patient samples, AUC sum is average of AUC and AUC one-leave-out.

Table S10. Summary of average AUC results for various substrates and various nanoparticles .

Number of PC components	Au_100nm_AuNPs	Au_60nm_AuNPs	Ag_100nm_AuNPs	Ag_100nm_AgNPs	Al_tape_100nm_AuNPs	Al_tape_60nm_AuNPs	Si_60nm_AuNPs
5	0.771	0.711	0.765	0.642	0.515	0.632	0.620
7	0.774	0.695	0.793	0.686	0.562	0.651	0.691
9	0.829	0.702	0.861	0.734	0.592	0.718	0.681
11	0.824	0.724	0.869	0.737	0.589	0.709	0.713
13	0.846	0.752	0.864	0.737	0.700	0.706	0.782
15	0.843	0.752	0.864	0.727	0.689	0.696	0.786
17	0.834	0.756	0.869	0.734	0.698	0.687	0.784
19	0.838	0.769	0.859	0.770	0.721	0.673	0.772
21	0.829	0.779	0.875	0.761	0.716	0.670	0.791
23	0.821	0.805	0.865	0.761	0.723	0.673	0.783
25	0.812	0.800	0.858	0.759	0.729	0.687	0.780
**max**	**0.846**	**0.805**	**0.875**	**0.770**	**0.729**	**0.718**	**0.791**
**relative performance**	**118**	**112**	**122**	**107**	**102**	**100**	**110**

The average AUC is the average of AUC and AUC one-leave- out.

Compassion of Cost: Commercial Gold Film substrates vs Commercial Silicon Wafer Substrates (January 2023)

Ted Pella sells ultra-flat 6 inch silicon wafers for 104.5 USD (28 inch 2 each), a piece for 1-9 pieces (product number 61015 at https://www.tedpella.com/vacuum_html/Substrates_Supports_Wafers_Slides.htm.aspx , or **3.73 USD / inch^2 of Si surface**. The least expensive gold-coated microscope slides from Ted Pella are 92.4 USD for each or **30.8 USD/inch^2** (product number 26002-G). Gold film on glass surface is 8.3 times more expensive than Si wafer surface, if bought from Ted Pella, USA, which is the most competitive price quote pair available in Kazakhstan to the best of our knowledge . Sigma Aldrich has several times more expensive substrates (both Si wafer (22 USD/inch^2) and Au film(60 USD/inch^2)) per inch^2. Before pandemic (fall 2019) the cost ratio per square inch of

gold to silicon wafer substrates for Ted Pella for the same products was about 11 (23.7 USD/inch^2 for gold film and 2.1 USD/inch^2 for silicon wafer) , which shows 30% and 78% price hike from late Fall 2019 to January 2023, making a clear demonstration of inflation (average 9 to 20+% annually for 3 years) in research related consumables and materials.

Overall, the cost of Si surface would be about one order of magnitude lower than the cost of gold film surface, but if NOT ultra-flat Si wafers but a bit lower grade of Si wafers is used, the Silicon substrates would be even less than 10 times costly as any commercially available gold coated substrate.

The cost of 60 nm commercial gold nanoparticles from sigma Aldrich is about 510 USD for 100 mL (467 euro)and we use about 16 × 0.015 mL = 0.24 mL of nanoparticles per inch^2. Therefore, the bottle can cover 100/0.24 = 417 inches^2 so the AuNP cost per inch^2 is 510/417 = 1.22 USD /inch^2 or 1.2 USD /inch^2

For 60 nm AuNPs+ Gold cost is 30.8+1.2 = 32 USD per inch and AuNPs +silicon 3.7+1.2 = 4.9 USD/inch^2

Conclusion In January 2023, when we calculated the cost of materials in Kazakhstan from same companies for both substrates, AuNPs@Gold film substrate is ×6.5 more expensive than AuNPs@Silicon (even on ultra-smooth silicon wafer)
